# Mobile Apps for Vaccination Services: Content Analysis and Quality Assessment

**DOI:** 10.2196/50364

**Published:** 2024-10-03

**Authors:** Chenchen Zhang, Xing Guo, Rui Zhu, Wenjie Hou, Lingmeng Wang, Fuzhi Wang, Li Zhang, Dan Luo

**Affiliations:** 1 School of Nursing Bengbu Medical College Bengbu China; 2 School of Health Administration Bengbu Medical College Bengbu China

**Keywords:** vaccination service app, Mobile Application Rating Scale, MARS, quality evaluation, app, apps, application, applications, quality, evaluation, rating, mHealth, mobile health, service, services, service content, user evaluation, vaccine, vaccines, public health

## Abstract

**Background:**

Vaccination services are increasingly in demand by the public, and mobile apps are an effective tool to meet that demand. However, the characteristics and quality of these apps are unknown.

**Objective:**

Commonly used vaccination service apps on the market were surveyed with regard to quality, service content, and user experience to evaluate and guide users.

**Methods:**

The Qimai Data mobile app data analytics platform was used to search for common vaccination service apps by keyword, and the WeChat and Alipay platforms were searched for apps. The apps included in the study were independently evaluated by two reviewers using the Mobile Application Rating Scale, and the service content and user experience of the apps were analyzed. The intragroup correlation coefficient between raters was used to measure interrater reliability.

**Results:**

In the app stores of the four major Android platforms and the iOS app store, 1092 and 207 apps were found, respectively; 189 WeChat applets and 30 Alipay applets were also found. A total of 29 apps was ultimately included in this study according to the inclusion criteria, including 21 independent apps, 4 WeChat applets, and 4 Alipay applets. Significant differences were found between independent apps and applets in terms of the quality score (*t*_449.57_=–5.301; *P*<.001) and the subjective quality score (*z*=–4.753; *P*<.001). No significant differences were found between iOS and Android platforms in terms of the quality score (*t*_1404_=–2.55; *P*=.80) and the subjective quality score (*z*=–0.137; *P*=.89). There was good intragroup consistency among the raters.

**Conclusions:**

In this study, independent apps and nonindependent apps that rely on social and payment platforms for implementation were included in the vaccination services category. The overall quality of these apps was acceptable. Nonindependent running apps were found to have slightly lower scores and showed room for improvement, and scores for the participatory apps were found to be generally low overall.

## Introduction

Vaccination is the most effective primary prevention for infectious diseases. At birth and while breastfeeding, infants can acquire antibodies against infectious diseases from their mothers, but as children grow older, the effectiveness of these antibodies gradually diminishes and disappears [1,2]. Therefore, a detailed immunization program has been developed for children by the Chinese government. It is important to maintain children’s health by ensuring that they receive the required vaccinations in a timely manner. The traditional vaccine consulting service model cannot ensure that the various indicators of immunization planning meet the national requirements [2]. Many preventive vaccination clinics are gradually implementing digital management, and the flow of information incorporates networked automated distribution management. The use of the internet and vaccination platforms presents a new model for preventive vaccination that is gradually being accepted by parents [3].

Since 2021, China has been the country with the largest number of smartphone users in the world, with more than 950 million [4]. The development of smartphones has increased the development of vaccination service apps. Vaccination units can send vaccination appointment information, notifications about necessary vaccines, and publicity and educational articles on preventive vaccination through apps, which can save the expenses of SMS text messaging notifications and publicity costs [5]. Children’s parents can keep abreast of their children’s vaccination status, vaccine appointment schedules, inventory information of the vaccination unit, online consultations on vaccination-related knowledge, independent booking of vaccination appointments, and other convenient and user-friendly services [6]. These apps are accessible, optimize the efficiency of services [7-9], enhance the motivation to vaccinate, and improve vaccination rates [7,10,11]. These findings are widely recognized in academia. In a needs assessment survey of vaccination service apps, participants expressed their interest in using such apps [12]. In a study by Zaidi et al [13], such apps were more operational, acceptable, and practical. Wei et al [14] found that parents of children using mobile apps for vaccine advocacy scored significantly higher in knowledge and trust than those who used traditional methods. To a certain extent, the vaccination capacity of the population has been enhanced by vaccination service apps [9]. While vaccination service apps are critical to increasing vaccination rates. The essential prerequisite is to ensure that they are of high-quality. A review of relevant studies on such apps reveals that past studies have mainly focused on service content and improving vaccination rates, and there is a lack of research related to quality assessment and user experience.

The Mobile Application Rating Scale (MARS) was developed by a multidisciplinary team of experts as a simple, objective, and reliable tool for researchers, developers, and health professionals to assess the quality of apps. The scale is widely used for apps for weight management [15], disease [16,17], mental health [18], and pain [19]. In China, mobile terminals consist of two main areas: independent apps and nonindependent apps that rely on social or payment platforms (Alipay applets and WeChat applets). Retrieval strategies were designed for each of these two types.

In this study, the commonly used vaccination service apps on the market were investigated, assessed for quality using MARS, and analyzed for service content and user ratings, assisting in the development and improvement of such apps and popularizing their use. At the same time, this information will also provide valuable suggestions for users when choosing apps.

## Methods

### App Search Strategy

Regarding independent apps, the iOS app store was searched using the keywords “vaccine,” “vaccine service,” “immunization,” and “vaccination.” Apps for Android phones were screened by entering the keywords “vaccine,” “vaccine service,” “immunization,” and “vaccination” on the website QiMai Data. The keywords “immunization” and “vaccination” were used to screen for apps on the Huawei, Xiaomi, OPPO, and VIVO smartphones. The top 50 apps found in the search that met the requirements were extracted and ranked according to the number of downloads. Data related to all apps in the App Store, Google Play, and nine major domestic Android marketplaces (Huawei, Xiaomi, OPPO, VIVO, Meizu, Baidu, 360 App Store, and Pea Pod) were provided by QiMai Data.

### Platform-Dependent Applets

WeChat applets were searched by entering the keywords “vaccine,” “vaccine service,” “immunization,” and “vaccination” in the WeChat search window. Alipay applets were searched by entering the keywords “vaccine,” “vaccine service,” “immunization,” and “vaccination” in the Alipay search window.

### App Filter

For the two different types of apps/applets with vaccination services, the type of functions were noted, and two sets of exclusion criteria were designed. For independent apps, the exclusion criteria were as follows: apps that were not related to the theme, apps that have not been updated for 1 year, apps that have been downloaded <100,000, apps that were not in simplified Chinese, apps with a rating of <2, and apps that cannot be downloaded and used normally. For platform-dependent applets, the exclusion criteria were as follows: applets that were not related to the theme, applets that did not work properly, applets on WeChat with <10,000 recent users, and applets on Alipay with <100,000 recent users.

### Assessment Tools

In this study, MARS was used to complete the evaluation of mobile apps. MARS includes five core components: engagement, functionality, aesthetics, information, and subjective quality. The rating scale was a 5-point scale: inadequate (1), poor (2), acceptable (3), good (4), and excellent (5). The subjective quality section comprised four subjective evaluation questions [20].

### Review Process

The review process consisted of three steps. In the first step, the basic description and technical information of these 29 apps were collected from the app stores (iOS and Android) and the Alipay and WeChat applet platforms in accordance with the requirements of the first part of MARS. In the second step, all reviewers studied the instructional video training provided by the MARS developers together [15] and discussed and reached a consensus on the content of the queries. The evaluation was performed by three reviewers with two apps; two WeChat applets and two Alipay applets were randomly selected for pre-evaluation. The scores were discussed to agree on the evaluation criteria as much as possible. In the third step, all 29 apps were installed on a phone (Android device: Xiaomi Mi11Lite, version MIUI13.0.12 stable version; iPhone device: iPad mini, version: 15.6). The quality of the included apps were evaluated using MARS. The evaluation was performed by two independent raters assessing the same app at the same time.

### User Experience

The qualitative analysis software NVivo 14.0 (Lumivero) was used for data entry and coding, which followed the process of grounded theory with open coding, spindle coding, and selective coding. Open coding refers to the process of discovering conceptual categories from the data and then naming and generalizing the phenomena under study; spindle coding refers to the process of establishing categories through inductive deduction on the open-coded categories; selective coding is the process of linking the core categories to other main categories around the core categories to construct a new theoretical framework in the form of a storyline [21]. The specific steps of this operation were as follows. First, one researcher completed the extraction and analysis of the basic categories and concepts, and another researcher conducted a theoretical protection test on the coding. In case of disagreement, a third person was invited to participate in the discussion to ensure that all conclusions were agreed upon. Grounded theory is a qualitative approach that emphasizes the generalization or emergence of information from data to build a theory or model [22].

### Statistical Analysis

Data were analyzed using descriptive and analytical statistics, with numerical variables describing the means and SDs and categorical variables describing the frequencies of use and percentages of market share. Data collection and collation were completed using Excel 2016 (Microsoft Corporation). Data analysis was conducted with SPSS Statistics version 26 (IBM Corp). The intragroup correlation coefficient was used to measure interrater agreement. A 1-way analysis of the apps on different platforms was performed using *t* tests and *Z* tests. *T* tests were used if the data conformed to a normal distribution; otherwise, the *Z* test was used. The correlation between app quality scores, reviews, and MARS quality scores was analyzed using Pearson correlation analysis.

## Results

### Overview

**Figure 1:**
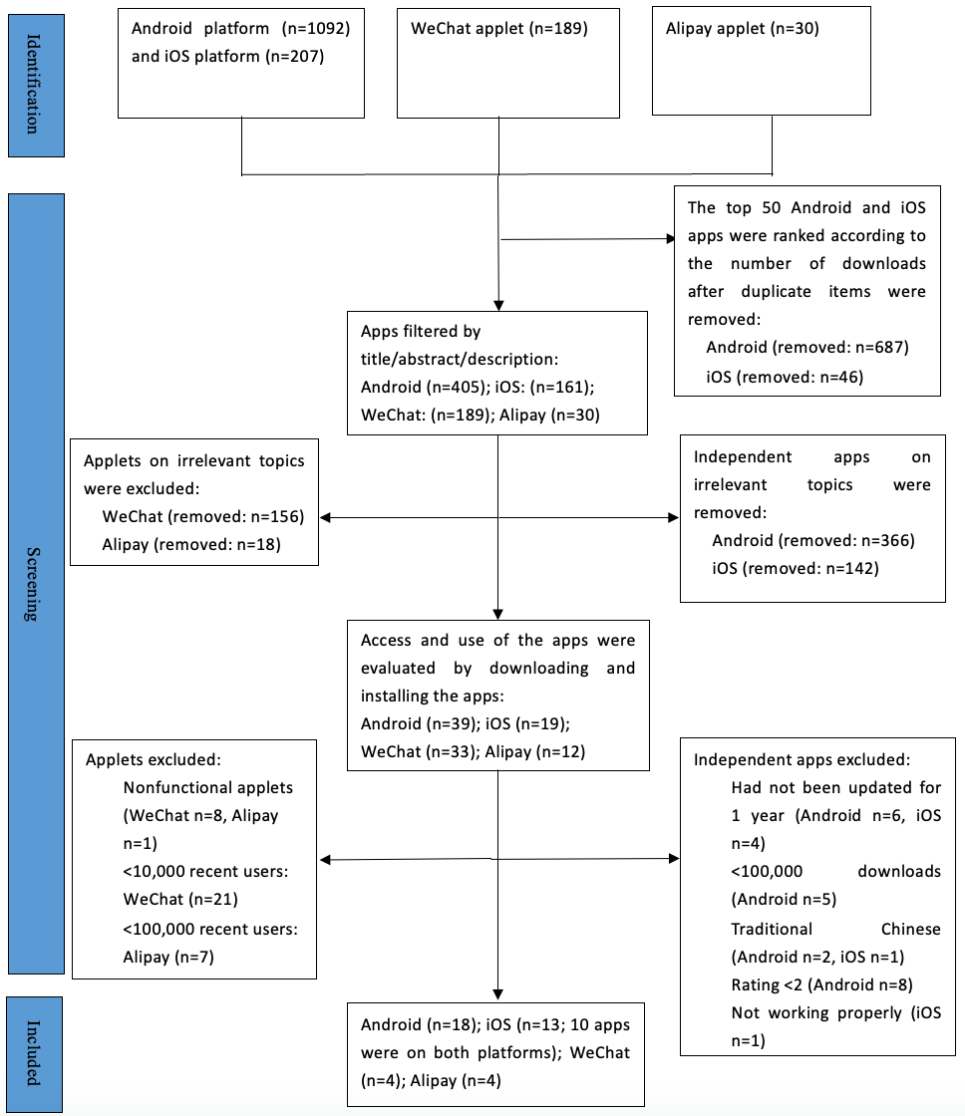
App screening flowchart.

A flowchart of the app screening is shown in Figure 1.

Of the 29 eligible apps, 21 were from app stores, 4 were Alipay applets, and 4 were WeChat applets. All apps were free to download, and the number of downloads and ratings were provided by the search platform.

The largest percentage of the apps with a user star rating of 4-5 were on Android (10/18, 56%), followed by iOS (6/13, 46%). Of the 18 apps for Android, 8 (44%) had more than 10 million downloads. Among the types of apps, the medical and sports health categories accounted for the largest proportion (11/31, 35.4% and 8/31, 25.9%, respectively), while the learning and education and convenient life categories accounted for the smallest proportion (both at 1/31, 3.2%; Table 1).

**Table 1:** Basic app characteristics.

Properties			Apps (N=39), n (%)
**Platform**			
	Apple		13 (33)
	Android		18 (46)
	Apple + Android		10 (26)
	Alipay applet		4 (10)
	WeChat applet		4 (10)
**Category**			
	Sports health		8 (26)
	Medical		11 (35)
	Medical health		5 (16)
	Health and fitness class		2 (7)
	Children’s class		3 (10)
	Learning and education class		1 (3)
	Convenient life class		1 (3)
**Downloads^a^ (Android: n=18)**			
	<999,999		3 (17)
	999,999-10,000,000		7 (39)
	>10,000,000		8 (44)
**User star rating^b^**			
	**Android (n=18)**		
		2.0-2.9	3 (17)
		3-3.9	5 (28)
		4-5	10 (56)
	**iOS (n=13)**		
		2.0-2.9	2 (15)
		3-3.9	5 (39)
		4-5	6 (46)

^a^No download descriptions were provided for iOS or the Alipay and WeChat applets.

^b^The Alipay and WeChat applets do not provide user star ratings.

### Service Content of the App

There were 10 categories covered by the apps: vaccination appointment management, vaccination record management, vaccination information service, internet hospital, science knowledge, drug administration, health management, family doctor, specialist consultation, and peer support and feedback. Among the 21 apps, the most used service on iOS was the science knowledge category (n=10, 48%), followed by the vaccination appointment management (n=9, 43%) and vaccination information service (n=8, 38%) categories, and the least used service was the health management category, followed by the family doctor and the specialist consultation categories (both n=1, 5%). The most used service on the Android platform was the science knowledge category (n=14, 67%), and the least used service was the vaccination record management category, followed by the health management, family doctor, specialist consultation, and peer support and feedback categories (all n=2, 10%). Among the 8 applets, WeChat applets accounted for the highest percentage of the vaccination appointment management, vaccination information service, and science knowledge categories (all n=4, 50%). Among the Alipay applets, the most often used service was the science knowledge category (n=4, 50%), while the vaccination record management, internet hospital, and health management categories accounted for the least used services (all n=1, 13%; Table 2).

**Table 2:** Service categories of the apps.

Categories	App (n=21), n (%)		Applet (n=8), n (%)	
	iOS	Android	WeChat	Alipay
Vaccination appointment management	9 (43)	11 (52)	4 (50)	3 (38)
Vaccination record management	2 (10)	2 (10)	1 (13)	1 (13)
Vaccination information service	8 (38)	13 (62)	4 (50)	3 (38)
Internet hospital	6 (29)	8 (38)	2 (25)	1 (13)
Science knowledge	10 (48)	14 (67)	4 (50)	4 (50)
Drug administration	5 (24)	7 (33)	0 (0)	0 (0)
Health management	1 (5)	2 (10)	2 (25)	1 (13)
Family doctor	1 (5)	2 (10)	0 (0)	0 (0)
Specialist consultation	1 (5)	2 (10)	0 (0)	0 (0)
Peer support and feedback	2 (5)	2 (10)	0 (0)	0 (0)

### MARS Quality Score

Two independent researchers calculated the scores for each part of all apps according to the MARS evaluation criteria, as detailed in Multimedia Appendices 1 and 2 and Table 3. The interrater reliability intragroup correlation coefficient for each component between the two MARS raters was 0.840 for participatory, 0.733 for functionality, 0.769 for aesthetics, 0.968 for information, 0.943 for app quality score, and 0.637 for subjective quality score, as detailed in Table 4. Significant differences were found between independent apps and applets in terms of quality score (*t*_449.57_=–5.301; *P*<.001) and subjective quality score (*z*=–4.753; *P*<.001). No significant differences were found between iOS and Android platforms in terms of quality score (*t*_1404_=–2.55; *P*=.80) and subjective quality score (*z*=–0.137; *P*=.89; Table 5).

Correlations between individual app quality scores, subjective quality scores, app ratings, and numbers of reviews were analyzed, and significant correlations were found between the app rating and number of reviews (*r*=0.364; *P*=.04), rating and subjective quality score (*r*=0.47; *P*=.006), and app quality score and subjective quality score (*r*=0.816; *P*<.001; Table 6).

**Table 3:** Applet quality rating scale.

Applet		Section A: participatory, mean (SD)	Section B: functionality, mean (SD)	Section C: aesthetics, mean (SD)	Section D: information, mean (SD)	App quality score, mean (SD)	Section E: subjective quality score, mean (SD)
**Alipay**							
	Vaccination Services	2.84 (0.48)	4.00 (0)	3.33 (0.52)	2.79 (1.37)	3.00 (1.07)	2.88 (1.07)
	Vaccination Quaicha	1.70 (0.48)	3.88 (0.35)	3.00 (0)	2.21 (1.58)	2.55 (1.27)	2.13 (0.64)
	Medical Health Channel	2.10 (0.57)	3.88 (0.35)	3.33 (0.52)	3.07 (1.49)	3.03 (1.05)	3.25 (0.46)
	XinYun Vaccination Inquiry	1.90 (0.57)	3.88 (0.35)	3.33 (0.52)	2.29 (1.64)	2.68 (1.30)	3.00 (0)
**WeChat**							
	Tengxun Health	2.10 (0.57)	4.00 (0)	3.67 (0.52)	2.93 (1.44)	3.05 (1.16)	3.38 (0.52)
	Rainbow Doctor	2.50 (0.71)	4.00 (0)	3.67 (0.52)	2.86 (1.41)	3.13 (1.09)	3.13 (0.35)
	Baby Plan Vaccination Assistant	2.10 (0.32)	3.88 (0.35)	3.83 (0.41)	2.93 (1.44)	3.05 (1.14)	3.13 (0.35)
	Shekangtong	2.10 (0.32)	3.88 (0.35)	3.50 (0.55)	2.93 (1.33)	3.00 (1.07)	3 (0)

**Table 4:** Interrater reliability table for each component of the two raters.

Quality	Rater 1, mean (SD)	Rater 2, mean (SD)	Intragroup correlation coefficient (95% CI)
Participatory	2.66 (0.73)	2.70 (0.74)	0.840 (0.785-0.882)
Functionality	4.13 (0.57)	4.19 (0.47)	0.733 (0.636-0.807)
Aesthetics	3.46 (0.52)	3.48 (0.52)	0.769 (0.667-0.843)
Information	3.00 (1.39)	3.00 (1.39)	0.968 (0.958-0.976)
App quality score	3.23 (1.11)	3.23 (1.11)	0.943 (0.933-0.952)
Subjective quality score	3.26 (0.62)	3.38 (0.64)	0.637 (0.513-0.735)

**Table 5:** Univariate analysis of the quality of apps based on the Mobile Application Rating Scale (MARS).

MARS quality evaluation			Score, mean (SD)	*t* test (*df*)	*P* value
**App quality score**				–5.301 (449.57)	<.001
	Applet		2.94 (1.16)		
	App		3.33 (1.07)		
	**Platform**			–2.550 (1404)	.80
		iOS	3.35 (1.10)		
		Android	3.34 (1.08)		
**Subjective quality score**				–4.753^a^	<.001
	Applet		2.98 (0.52)		
	App		3.41 (0.63)		
	**Platform**			–0.137^a^	.89
		iOS	3.45 (0.650)		
		Android	3.46 (0.637)		

^a^*Z* score.

**Table 6:** Correlation analysis of ratings, reviews, and Mobile Application Rating Scale quality scores.

		Number of reviews	App quality score	Subjective quality score
**Rating**				
	*r*	0.364	0.348	0.478
	*P* value	.04	.06	.006
**Number of reviews**				
	*r*	—^a^	0.230	0.155
	*P* value	—	.21	.40
**App quality score**				
	*r*	0.230	—	0.816
	*P* value	.21	—	<.001

^a^Not applicable.

### User Experience

The first 20 reviews of each app were analyzed, and grounded theory was used to analyze, classify, and code the reviews. The user concerns were divided into five topics: content, functionality, experience, service attitude, and privacy. In general, users who rated the first four aspects were partly satisfied and partly dissatisfied. However, users who rated privacy were not satisfied.

Users who evaluated the content gave satisfactory ratings for rich medical knowledge, while dissatisfaction was expressed for lost user data and vaccination record inquiries.

Quite useful, you can acquire vaccination-related knowledge...With this software, it's more convenient for my baby to get vaccinated.

N1

The new page is too cluttered...dense with words, the block is not obvious...There is no desire to see, floating ads are also an eyesore.

N3

Users who rated the app in terms of functionality gave poor ratings to the software’s forced updates, flashbacks, and customer service features, and were satisfied with the app’s ability to make vaccination appointments and provide vaccination knowledge.

Forced to update, nonstop flashbacks, point in and flashback...cannot access any feature.

N4

The product did not go online after rigorous testing, as a software test engineer seeing this product, I really want to laugh...There were a lot of mistakes when using the software, and even the basic master rod flow could not be used.

N5

In terms of the experience, some patients found the app to be very helpful. However, there was the problem of unreliable registered hospitals.

The user experience is very good, online consultation is very convenient, medical examination appointment, medical examination report view, report interpretation, etc...experience is very good.

N6

Poor experience, please take this software down, don't let it cheat people. The customer service inside seems to be the same, just as fake...no one responded when I spoke to them, and there was no refund page when it came time to refund.

N7

For users of the service attitude evaluation, some questioned the accuracy and quality of the response to questions, while some thought the doctor was professional and quick to review their queries.

The doctor’s response is slow and perfunctory...No one answers after half a day of waiting, the response is not timely even if the question asked is not detailed... and the charge is not refundable.

N8

Very efficient, there are three opportunities to ask questions... doctor will also give you an answer, give the appropriate explanation, and make a clearer diagnosis later. Very efficient.

N9

Users said they were not satisfied with the privacy of the evaluation because the software needed too many permissions, and they were concerned for the security of their personal information.

Leakage of personal information...did not place an order, received an SMS notification of a refund, customer service is difficult to deal with and is delinquent in intervening.

N10

The information security of the user is not well done, the child vaccination hospital has inexplicably become other places, and my vaccination records have been altered.

N11

## Discussion

### Principal Findings

In this study, commonly used vaccination service apps on the market were investigated, assessed for quality using MARS, and analyzed for service content and user ratings. The results found the apps to be of good overall quality, with no significant differences between the iOS and Android platforms. This study also found that the apps were classified according to their service content into the following categories: vaccination appointment management, vaccination information service, vaccination record management, internet hospital, science knowledge, drug administration, health management, family doctor, specialist consultation, and peer support and feedback. The service with the highest frequency of use was scientific knowledge. Users were mainly concerned with five issues in the use of this type of app: content, functionality, experience, privacy, and service attitude. The majority of users were not satisfied with the privacy aspects of the apps.

A total of 29 apps were ultimately evaluated in-depth through a search and screening of over 1000 apps. The largest percentage of apps with a user star rating of 4-5 reflects, to some extent, that most users were satisfied with such apps. For Android, 44% of the apps had more than 10 million downloads, which also indicates the popularity of the apps.

### MARS Quality Score

In this study, MARS was used as a quality assessment tool. Based on a comprehensive assessment of apps using MARS, Android and iOS were found to be nonsignificantly different in terms of app scores, with overall good quality but room for improvement. Overall, this type of app scored highest in functionality but not in engagement. Therefore, developers are advised to focus on meeting users’ needs in all aspects of functionality, multidimensionality, and depth while also paying attention to the design of the app in terms of entertainment, fun, interactivity, and other engagement aspects. Regarding the evidence base category in the scale, among the apps, only the Xiaodou Miao app was validated by evidence in published scientific literature, while the remaining apps scored 0 in this category. This suggests that there was a lack of studies validating these apps and that in-depth studies should be conducted in this area in the future.

### Service Content of the App

The apps were classified according to their service content into vaccination appointment management, vaccination information service, vaccination record management, internet hospital, science knowledge, drug administration, health management, family doctor, specialist consultation, and peer support and feedback categories. Among the independent apps, the most frequently used was the science knowledge category, which all the applets offered. To a certain extent, this shows that such apps attach more importance to the popularization of scientific knowledge. Vaccination appointment management was considered very convenient by parents of young children because of the reduced waiting time [23], bearing in mind that low vaccination rates among young children were partly due to parental indecision [24]. Studies have shown that educational interventions for vaccination-hesitant parents can increase vaccination coverage in children aged 6 months to 6 years [25], which explains the importance that developers place on providing scientific knowledge and that health interventions for users based on scientific evidence play a positive role in increasing immunization coverage. When the app ratings were analyzed in relation to the number of reviews, they were found to be moderately correlated, indicating that higher-rated apps were more popular among users. User feedback was used by developers to gain insight into the reality of app use and thus guide future development and updates.

### User Experience

User evaluations of the apps expressed the real experience of using the apps. Users were concerned about five main issues: content, functionality, experience, privacy, and service attitude. Users who rated the privacy of the app were dissatisfied. App developers should focus on privacy aspects in future improvements and the development of high-quality apps. Additionally, compared with the platform-dependent applets, the user experience was better on the independent apps due to the higher security, the information being updated, and the strict review of the app stores.

### Limitations

This study has several limitations. First, only the four most popular Android phone brands and related apps for iOS were evaluated, and only free Chinese apps from a specific period were included. Second, the researchers evaluated the apps based on short-term use, and some apps were excluded for reasons such as the inability to open or the need to provide an internal institutional registration code, so the findings may have been selectively biased. At present, versions of MARS have been developed and validated in Germany, Turkey, and Korea [26-28]. However, there were no scales or validation studies suitable for China. Last, given the current status of vaccination for COVID-19 in China, such apps were excluded due to the strong user or time-sensitive nature of ad hoc apps that provide only COVID-19 vaccination appointment services, which may also have had an impact on our results.

### Conclusions

In this study, independent apps and nonindependent apps that rely on social or payment platforms (Alipay and WeChat applets) were included in the vaccination service category. The overall quality of such apps was considered acceptable, but the nonindependent apps were rated slightly lower, with room for improvement.

## Appendix

Multimedia Appendix 1 App quality rating scale.

Multimedia Appendix 2 Mobile Application Rating Scale scores for all apps.

## Abbreviations

MARS: Mobile Application Rating Scale
