# Supplementary material for: Mobile Apps for Vaccination Services: Content Analysis and Quality Assessment
Source: Online J Public Health Inform. 2024 Oct 3;16:e50364. doi: 10.2196/50364 (PMC11487208; doi:10.2196/50364)
Supplement: Multimedia Appendix 1 [file ojphi_v16i1e50364_app1.docx]

| **App Name** | **Section A:**  **Participatory** | | **Section B:**  **Functionality** | |  |  | **Section C:**  **Aesthetics** | | **Section D:**  **Information** | | **Application quality score** | | **Section E：**  **Subjective quality score** | | |
| --- | --- | --- | --- | --- | --- | --- | --- | --- | --- | --- | --- | --- | --- | --- | --- |
|  | iOS | Android | iOS | Android |  |  | iOS | Android | iOS | Android | iOS | Android | iOS | Android |  |
| Baby Notes | 3.80±0.42 |  | 4.50±0.53 |  |  |  | 3.67±0.52 |  | 3.21±1.58 |  | 3.71±1.11 |  | 4.13±0.64 |  |  |
| Rainbow Doctor | 2.80±0.79 |  | 4.50±0.53 |  |  |  | 3.67±0.52 |  | 2.86±1.41 |  | 3.32±1.19 |  | 3.35±0.46 |  |  |
| Baby Time | 2.90±0.57 |  | 4.38±0.74 |  |  |  | 3.33±0.52 |  | 3.00±1.52 |  | 3.32±1.16 |  | 3.25±0.46 |  |  |
| BlueCard.com |  | 2.40±0.52 |  | 4.00±0.76 |  |  |  | 3.33±0.52 |  | 3.14±1.41 |  | 3.16±1.10 |  | 3.25±0.46 |  |
| Dr. Chunyu |  | 2.60±0.52 |  | 4.25±0.46 |  |  |  | 3.33±0.52 |  | 3.29±1.44 |  | 3.32±1.09 |  | 3.50±0.53 |  |
| Dingxiang Mom |  | 2.80±0.42 |  | 4.00±0.76 |  |  |  | 3.33±0.52 |  | 3.21±1.42 |  | 3.29±1.04 |  | 3.63±0.52 |  |
| Health Taicang |  | 2.40±0.52 |  | 4.50±0.53 |  |  |  | 3.33±0.52 |  | 3.00±1.36 |  | 3.21±1.17 |  | 3.00 |  |
| Han Mom |  | 3.40±0.52 |  | 4.13±0.35 |  |  |  | 3.50±0.55 |  | 3.50±1.41 |  | 3.61±1.00 |  | 4.13±0.35 |  |
| Baby Cloud |  | 3.40±0.52 |  | 4.13±0.35 |  |  |  | 3.50±0.55 |  | 3.50±1.51 |  | 3.61±1.03 |  | 4.13±0.35 |  |
| Baby Parenting Album |  | 3.20±0.42 |  | 4.13±0.35 |  |  |  | 3.83±0.41 |  | 3.21±1.42 |  | 3.50±0.98 |  | 3.13±0.35 |  |
| Capital Vaccination Service |  | 2.40±0.52 |  | 4.50±0.52 |  |  |  | 3.33±0.52 |  | 2.71±1.20 |  | 3.11±1.13 | 3.13±0.35 | 3.13±0.35 |  |
| YueMiao | 2.40±0.25 | 2.50±0.53 | 3.75±0.89 | 3.75±0.89 |  |  | 2.67±0.52 | 2.67±0.52 | 2.57±1.09 | 2.57±1.09 | 2.79±0.96 | 2.82±0.95 | 2.75±0.71 | 2.88±0.64 |  |
| YiLu | 2.60±0.52 | 2.60±0.52 | 4.50±0.53 | 4.50±0.53 |  |  | 3.67±0.52 | 3.67±0.52 | 2.86±1.41 | 3.00±1.36 | 3.26±1.18 | 3.32±1.14 | 2.75±0.71 | 3.25±0.46 |  |
| XiaodouMiao | 3.80±0.63 | 3.50±0.53 | 4.13±0.35 | 4.50±0.53 |  |  | 3.67±0.52 | 4.00 | 3.86±0.53 | 4.00±0.55 | 3.87±0.53 | 4.00±0.53 | 4.13±0.50 | 3.97±0.59 |  |
| Aikang | 3.10±0.74 | 3.10±0.74 | 4.38±0.52 | 4.38±0.52 |  |  | 3.67±0.52 | 3.67±0.52 | 3.07±1.38 | 3.07±1.38 | 3.45±1.08 | 3.45±1.08 | 3.50±0.53 | 3.50±0.53 |  |
| YueMiao | 2.60±0.52 | 2.60±0.52 | 4.25±0.46 | 4.50±0.53 |  |  | 3.33±0.52 | 3.33±0.52 | 2.86±1.41 | 2.71±1.20 | 3.16±1.10 | 3.32±1.14 | 3.38±0.52 | 3.38±0.52 |  |
| Xiaohe Health | 3.20±0.63 | 2.80±0.42 | 4.25±0.46 | 4.25±0.46 |  |  | 3.67±0.52 | 3.67±0.52 | 3.00±1.47 | 3.14±1.41 | 3.42±1.08 | 3.37±1.05 | 3.38±0.52 | 3.25±0.46 |  |
| Tengxun  Yidian | 2.50±0.53 | 2.40±0.52 | 4.00±0.53 | 4.25±0.46 |  |  | 3.33±0.52 | 3.33±0.52 | 2.86±1.41 | 2.86±1.29 | 3.08±1.08 | 3.11±1.09 | 3.38±0.52 | 3.00±0.53 |  |
| Qingmiao Bao | 2.40±0.52 | 2.40±0.52 | 4.00 | 4.00 |  |  | 3.00 | 3.00 | 2.57±1.09 | 2.57±1.09 | 2.89±0.92 | 2.89±0.92 | 3.00±0.76 | 3.00±0.53 |  |
| Jingdong  Health | 3.40±0.52 | 3.20±0.42 | 4.38±0.52 | 4.25±0.46 |  |  | 3.50±0.55 | 3.33±0.52 | 3.07±1.38 | 3.00±1.36 | 3.50±1.03 | 3.37±1.00 | 3.63±0.52 | 3.38±0.74 |  |
| Dr. Ding Xiang | 3.20±0.42 | 3.20±0.42 | 4.38±0.74 | 4.63±0.52 |  |  | 3.67±0.52 | 3.67±0.52 | 3.21±1.48 | 3.29±1.44 | 3.53±1.08 | 3.61±1.08 | 3.38±0.52 | 3.38±0.52 |  |
