# Supplementary material for: Mobile Apps for Vaccination Services: Content Analysis and Quality Assessment
Source: Online J Public Health Inform. 2024 Oct 3;16:e50364. doi: 10.2196/50364 (PMC11487208; doi:10.2196/50364)
Supplement: Multimedia Appendix 2 [file ojphi_v16i1e50364_app2.pdf]

APP	Entertainment	Interest	SECTION A<br>Customisation	Interactivity
XiaodouMiao	3	4	4	3
	3	4	5	4
Aikang	3	3	3	2
	4	4	3	2
Xiaohe Health	3	3	3	2
	3	4	3	4
Jingdong Health	3	3	4	3
	4	4	4	3
Tengxun Yidian	2	2	3	2
	2	3	3	2
Dr. Ding Xiang	3	4	3	3
	3	4	3	3
Qingmiao Bao	2	2	3	2
	2	2	3	2
Baby Notes	4	4	3	4
	4	4	3	4
Rainbow Doctor	2	2	3	4
	2	2	3	4
Baby Time	2	3	3	3
	2	3	3	3
YiLu	2	3	3	2
	2	3	3	2
YueMiao	2	2	3	2
	2	2	3	2
YueMiao	2	3	3	2
	2	3	3	2
Android YueMiao	2	2	3	2
	2	2	3	3
YiLu	2	3	3	2
	2	3	3	2
XiaodouMiao	3	4	4	3
	3	4	4	3
Capital Vaccination Service	2	2	3	2
	2	2	3	2
YueMiao	2	3	3	2
	2	3	3	2
Xiaohe Health	3	3	3	2
	3	3	3	2
Tengxun Yidian	2	2	3	2
	2	2	3	2
Qingmiao Bao	2	2	3	2
	2	2	3	2
Jingdong Health	3	3	4	3
	3	3	4	3
Dr. Ding Xiang	3	4	3	3

	3	4	3	3
BlueCard.com	2	2	3	2
	2	2	3	2
Dr. Chunyu	2	3	3	2
	2	3	3	2
Dingxiang Mom	2	3	3	3
	2	3	3	3
Health Taicang	2	2	3	2
	2	2	3	2
Han Mom	3	3	4	3
	3	3	4	3
Baby Cloud	3	3	4	3
	3	3	3	4
Baby Parenting Album	3	3	3	3
	3	3	3	3
AppletVaccination Servi	2	2	3	2
	2	2	2	2
Vaccination Quaicha	2	2	2	1
	2	2	2	2
Medical Health Channel	2	2	2	1
	2	2	2	2
XinYun Vaccination Inquiry	2	2	2	1
	2	2	2	1
Tengxun Health	2	2	2	1
	2	2	2	2
Rainbow Doctor	2	2	2	4
	2	2	2	3
Baby Plan Vaccination Assista	2	2	2	2
	2	2	2	2
Shekangtong	2	2	2	2
	2	2	2	2

SECTION B

Target group	Performance	Ease of use	Navigation	Gestural design
4	5	4	4	4
4	4	4	4	4
3	5	4	4	4
4	5	4	5	4
3	5	4	4	4
4	4	4	5	4
3	5	4	4	4
3	5	4	5	4
3	5	4	4	4
3	4	4	4	3
3	5	5	4	4
3	5	5	4	3
3	4	4	4	4
3	4	4	4	4
4	5	5	4	4
4	5	5	4	4
3	5	5	4	4
3	5	5	4	4
4	5	5	4	4
3	5	5	3	4
3	5	5	4	4
3	5	5	4	4
3	5	4	3	3
3	5	4	3	3
3	4	5	4	4
3	4	5	4	4
3	5	4	3	3
3	5	4	3	3
3	5	5	4	4
3	5	5	4	4
4	5	5	4	4
3	5	5	4	4
3	5	5	4	4
3	5	5	4	4
3	5	5	4	4
3	5	5	4	4
3	5	4	4	4
3	5	4	4	4
3	5	4	4	4
3	5	4	4	4
3	4	4	4	4
3	4	4	4	4
3	5	4	4	4
3	5	4	4	4
3	5	5	4	4

3	5	5	4	5
3	5	4	3	4
3	5	4	3	4
3	5	4	4	4
3	5	4	4	4
3	5	4	3	4
3	5	4	3	4
3	5	5	4	4
3	5	5	4	4
4	5	4	4	4
4	4	4	4	4
4	5	4	4	4
4	5	4	4	4
4	5	4	4	4
4	4	4	4	4
3	4	4	4	4
3	4	4	4	4
1	4	4	4	4
1	4	4	3	4
3	4	4	4	4
3	3	4	4	4
3	4	4	4	4
2	3	4	4	4
3	4	4	4	4
3	4	4	4	4
3	4	4	4	4
3	4	4	4	4
3	4	4	4	4
2	4	4	3	4
3	4	4	4	4
2	3	4	4	4

SECTION C

Layout	Graphics	Visual appeal	Accuracy of app description
4	4	4	4
3	4	3	3
4	4	3	4
3	4	4	3
4	4	3	4
3	4	4	4
4	3	3	4
4	4	3	4
3	4	3	4
3	4	3	4
4	4	3	4
4	4	3	4
3	3	3	3
3	3	3	3
4	4	3	4
4	4	3	4
4	4	3	4
4	4	3	4
3	4	3	4
3	4	3	4
4	4	3	4
4	4	3	4
2	3	3	3
2	3	3	3
3	4	3	4
3	4	3	4
2	3	3	3
2	3	3	3
4	4	3	4
4	4	3	4
4	4	4	4
4	4	4	4
3	4	3	3
3	4	3	3
4	4	3	4
4	4	3	4
4	4	3	4
4	4	3	4
3	4	3	4
3	4	3	4
3	3	3	3
3	3	3	3
4	3	3	4
4	3	3	4
4	4	3	4

4	4	3	4
3	4	3	4
3	4	3	4
3	4	3	4
3	4	3	4
4	3	3	4
4	3	3	4
4	3	3	4
4	3	3	4
4	4	3	5
3	4	3	4
4	4	3	5
4	3	4	4
4	4	3	4
4	4	4	4
3	4	3	4
3	4	3	3
3	3	3	3
3	3	3	4
4	3	3	4
4	3	3	4
4	3	3	4
4	3	3	4
4	4	3	4
4	4	3	4
4	4	3	4
4	4	3	4
4	4	3	4
4	4	4	4
4	4	3	3
3	4	3	3

SECTION D

Goals	Quality of information	Quality of information
5	4	4
4	4	4
4	3	4
4	3	4
4	3	4
4	3	4
3	3	4
4	3	4
3	3	3
4	3	4
4	3	4
4	3	4
3	3	3
3	3	3
5	4	4
4	4	4
4	3	3
4	3	3
4	3	3
4	5	3
4	3	3
4	3	3
3	3	3
3	3	3
4	3	3
4	3	3
3	3	3
3	3	3
4	3	3
4	3	3
5	4	4
5	4	4
3	3	3
3	3	3
4	3	3
4	3	3
4	3	4
4	3	4
3	3	3
3	3	3
3	3	3
3	3	3
3	3	4
3	3	4
4	4	4

4	4	4
4	3	4
4	3	4
4	4	4
4	4	4
4	3	4
4	4	4
4	3	3
4	3	3
4	4	4
4	4	4
4	4	4
4	4	4
4	4	4
3	4	4
3	3	4
3	3	4
4	3	3
4	3	3
4	3	4
4	4	4
4	3	3
4	3	3
4	3	4
4	3	4
4	3	3
4	3	3
4	3	3
4	4	3
3	3	3
3	3	4

				SECT
Visual information	Credibility	Evidence base		App subjectiv
4	3	4	4	4
4	3	4	4	3
4	3	0	3	3
4	3	0	4	4
4	2	0	3	3
4	2	0	4	3
4	3	0	3	4
4	3	0	4	4
4	2	0	3	3
4	2	0	4	4
4	3	0	3	4
5	3	0	3	3
3	3	0	3	2
3	3	0	4	2
4	2	0	4	4
4	2	0	3	4
4	2	0	3	3
4	2	0	3	4
4	2	0	3	3
4	2	0	3	4
4	2	0	3	3
4	2	0	3	3
3	3	0	2	3
3	3	0	2	2
4	2	0	3	3
4	2	0	3	3
3	3	0	2	3
3	3	0	2	3
4	3	0	3	3
4	3	0	3	3
4	3	4	4	4
4	3	4	4	3
4	3	0	3	3
4	3	0	4	3
4	3	0	3	3
4	3	0	3	4
4	3	0	3	3
4	3	0	3	3
4	3	0	3	3
4	3	0	4	3
3	3	0	3	2
3	3	0	4	3
4	3	0	3	4
4	3	0	2	4
4	3	0	3	4

4	3	0	3	3
4	3	0	3	3
4	3	0	3	4
4	3	0	4	4
4	3	0	3	4
4	3	0	3	4
4	3	0	4	4
3	4	0	3	3
3	4	0	3	3
4	4	0	4	4
4	4	0	4	4
4	3	0	4	4
3	4	0	4	5
4	3	0	3	3
4	3	0	4	3
4	2	0	3	3
4	2	0	3	3
0	2	0	2	2
0	2	0	2	2
4	2	0	3	3
4	2	0	3	3
0	2	0	3	3
0	2	0	3	3
4	2	0	4	3
3	2	0	4	3
4	2	0	3	3
4	2	0	4	3
4	2	0	3	3
4	2	0	3	4
4	4	0	3	3
4	4	0	3	3

ION E

e quality Score

5	4
5	4
3	4
3	4
3	4
3	4
3	4
3	4
3	3
3	4
3	4
3	4
3	3
3	4
5	4
5	4
3	3
4	3
3	3
3	4
3	4
4	4
3	3
3	4
3	4
4	4
3	3
3	4
3	4
3	4
5	4
4	4
3	3
3	3
3	4
3	4
3	4
3	4
3	3
3	3
3	3
3	3
3	4
3	4
3	4

3	4
3	3
4	3
3	4
3	3
3	4
3	4
3	3
3	3
5	4
4	4
5	4
5	4
3	3
3	3
3	3
2	3
1	3
2	3
3	4
3	4
3	3
3	3
3	4
3	3
3	3
3	3
3	3
3	3
3	3
3	3
